# Detecting horizontal gene transfer with metagenomics co-barcoding sequencing

**DOI:** 10.1128/spectrum.03602-23

**Published:** 2024-02-05

**Authors:** Kai Han, Jiarui Li, Duo Yang, Qinghui Zhuang, Hui Zeng, Chengbo Rong, Jinglin Yue, Na Li, Chaoyang Gu, Liang Chen, Chen Chen

**Affiliations:** 1 Biomedical Innovation Center and Beijing Key Laboratory for Therapeutic Cancer Vaccines, Beijing Shijitan Hospital, Capital Medical University, Beijing, China; State Key Laboratory of Food Science and Resources, Nanchang, China

**Keywords:** horizontal gene transfer, metagenomics co-barcode sequencing, multi-drug resistant bacteria, evolution

## Abstract

Horizontal gene transfer (HGT) is the process through which genetic information is transferred between different genomes and that played a crucial role in bacterial evolution. HGT can enable bacteria to rapidly acquire antibiotic resistance and bacteria that have acquired resistance is spreading within the microbiome. Conventional methods of characterizing HGT patterns include short-read metagenomic sequencing (short-reads mNGS), long-read sequencing, and single-cell sequencing. These approaches present several limitations, such as short-read fragments, high amounts of input DNA, and sequencing costs, respectively. Here, we attempt to circumvent present limitations to detect HGT by developing a metagenomics co-barcode sequencing workflow (MECOS) and applying it to the human and mouse gut microbiomes. In addition to that, we have over 10-fold increased contig length compared to short-reads mNGS; we also obtained exceeding 30 million paired reads with co-barcode information. Applying the novel bioinformatic pipeline, we integrated this co-barcoding information and the context information from long reads, and observed over 50-fold HGT events after we corrected the potential wrong HGT events. Specifically, we detected approximately 3,000 HGT blocks in individual samples, encompassing ~6,000 genes and ~100 taxonomic groups, including loci conferring tetracycline resistance through ribosomal protection. MECOS provides a valuable tool for investigating HGT and advance our understanding on the evolution of natural microbial communities within hosts.

**IMPORTANCE:**

In this study, to better identify horizontal gene transfer (HGT) in individual samples, we introduce a new co-barcoding sequencing system called metagenomics co-barcoding sequencing (MECOS), which has three significant improvements: (i) long DNA fragment extraction, (ii) a special transposome insertion, (iii) hybridization of DNA to barcode beads, and (4) an integrated bioinformatic pipeline. Using our approach, we have over 10-fold increased contig length compared to short-reads mNGS, and observed over 50-fold HGT events after we corrected the potential wrong HGT events. Our results indicate the presence of approximately 3,000 HGT blocks, involving roughly 6,000 genes and 100 taxonomic groups in individual samples. Notably, these HGT events are predominantly enriched in genes that confer tetracycline resistance via ribosomal protection. MECOS is a useful tool for investigating HGT and the evolution of natural microbial communities within hosts, thereby advancing our understanding of microbial ecology and evolution.

## INTRODUCTION

Horizontal gene transfer (HGT) is a fundamental process through which bacteria exchange genetic information. HGT has been reported in genes associated with antibiotic resistance, virulence, and metabolic functions (1, 2) . The transfer of antibiotic resistance genes through HGT currently represents a major public health concern due to the potential emergence of antibiotic-resistant bacterial strains and reduced efficacy of available antibiotics for treating bacterial infections (3–5). Accordingly, understanding the mechanisms of HGT and the factors influencing its frequency and directionality can aid the development of preventive strategies to the spread of antibiotic resistance.

The development of sequencing techniques facilitated the genetic study of microorganisms and enabled genome assembly and the identification of HGT events. At present, there are three main sequencing methods routinely employed, including short-reads metagenomic next-generation sequencing (short-reads mNGS), long-read sequencing, and single-cell sequencing (6). The short-reads mNGS technique directly enables sequencing all genomes present in a microbial sample and provides a better taxonomic resolution and more comprehensive genomic information. Importantly, this technique is also cost-effective and produces reads with low error rates (7, 8). However, short reads limit the study of repeat sequences in the genome and highly similar regions of different strains or species due to mapping issues and fragmented genome assemblies (8–10), hampering genome completeness (11) and making it unsuitable for HGT detection. In contrast, long reads such as those produced by Oxford Nanopore Technology and Pacific Biosciences (PacBio) improve genome assembly and make it possible to obtain more contiguous sequences (12–15). However, these techniques require high DNA inputs that are typically not available for microbiome research. Finally, single-cell sequencing (16–18) separates genome sequences with the least contamination, but is also limited by several factors, such as the lack of of suitable commercial products, complex and time-consuming experimental procedures, the need for high-quality samples, and high associated costs (19).

Here, we present a new metagenomics co-barcoding sequencing (MECOS) system that has the capability to acquire long fragment information and a large count co-barcode information to better identify HGT. Importantly, the sample input is small and the experimental process is comparably simple, circumventing the limitations of prevailing sequencing technologies. We sequenced microbial community standard and newly collected samples (three healthy human and three healthy mice gut microbiome samples) using short-reads mNGS and MECOS. Our results clearly demonstrate that MECOS offers higher genome assembly quality that enabled us to identify HGT from the contigs of each sample and associated antibiotic resistance genes. Accordingly, MECOS provides a novel approach for HGT detection in clinical samples.

## RESULTS

### Co-barcoding of long DNA fragments and MECOS workflow

**Fig 1:**
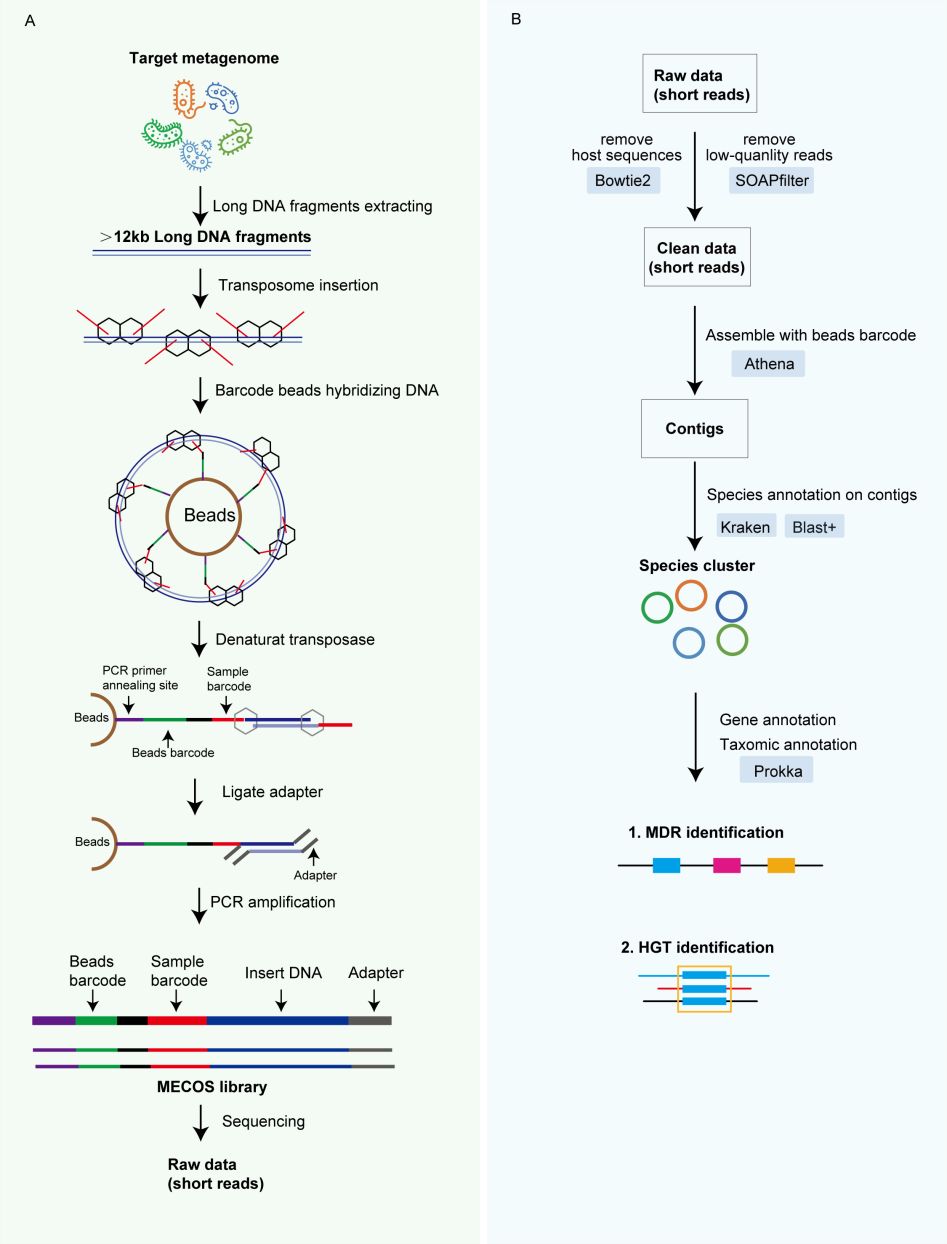
Overview of the MECOS sequencing and assembly approach. (**A**) Library preparation process. The DNA is extracted from microbiome samples and size-selected to enrich for long DNA fragments, then the following experimental process will be conducted: (i) transposome insertion, (ii) co-barcoding of long fragments, (iii) ligation and PCR, (iv) DNB generation and sequencing. Each library was labeled with a unique long-fragment barcode. (B) Bioinformatics analysis on co-barcoded reads: (i) removing host sequences and low quality reads, (ii) Athena assembling, (iii) species annotation, and (iv) gene annotation.

We constructed a transposome-barcoding scheme with linear amplification for metagenomic sequencing (Fig. 1A). Our approach mainly improved available technologies by (i) extracting long DNA fragments from microbiome, (ii) inserting a special transposome, (iii) utilizing barcode beads hybridizing DNA, and (iv) an integrated bioinformatic pipeline. Considering the different capsules present in bacteria, we used lysozyme to lyse bacteria and enriched for long DNA fragments using magnetic beads. We then used a special transposome, which is actually composed of two transposases and two transposon sequences, that is able to insert two known transposon sequences across each long DNA fragment, which is stabilized by the action of the transposases. The long DNA fragments that integrated the transposome were mixed with barcode beads, resulting in a library containing 30 million unique barcodes. Each barcode surface contained 400,000 copies of the same barcode, which were specific to the different beads. After removing the transposase, the long DNA sequences were fragmented into smaller pieces, each carrying identical barcodes. Then the co-barcoded reads were processed with bioinformatics analysis (Fig. 1B) . We note that each original long DNA fragment contained unique barcodes that can be utilized to assemble the reads and generate longer contigs.

We note that it is imperative to maintain a balanced ratio of barcode beads and long DNA fragments in the system, as an excess of the latter could result in multiple hybridizations of distinct long DNA fragments with a single barcode bead. This could, in turn, lead to different molecules containing the same barcode and affect downstream analyses. In contrast, an excess of barcode beads could result in specific long DNA fragments being labeled with multiple barcodes, negatively impacting assembly efficiency. Here, the ratio of barcode beads to long DNA fragments was maintained at approximately 5:1 to 3:1, ensuring that approximately 85% of barcode beads bound to a single specific DNA molecule.

### MECOS produces longer contigs for individual bacterial species

We employed MECOS on the Microbial Community Standard to assess the ability of MECOS to produce contigs. This analysis yielded a total of 101 M reads. We first used the Microbial Community Standard to evaluate whether MECOS could result in compositional and frequency biases on the bacterial community. The standard sample contained eight bacterial species, and we found that both MECOS and short-reads mNGS accurately reduced species composition, the community composition and abundance were largely concordant between MECOS and short-reads mNGS (Fig. S1A), and the relative read abundance between the methods was strong correlation (Fig. S1B). Then, we assigned a similar total length of contigs to each species (Fig. S1C). We note the N50 of each species was longer using MECOS (Fig. S1D) (Table 1).

**TABLE 1:** Basic sequencing data statistics and assembly of the Microbial Community Standard results

	*Bacillus subtilis*		*Enterococcus faecalis*		*Escherichia coli*	
	Short-reads mNGS	MECOS	Short-reads mNGS	MECOS	Short-reads mNGS	MECOS
Total contig length (Mb)	4.0	4.0	2.8	2.8	1.8	4.9
Contig number (≥200 k)	15	8	9	14	5	17
N50 contig length (kb)	294	1,897	256	618	108	114

	*Lactobacillus fermentum*		*Listeria monocytogenes*		*Pseudomonas aeruginosa*	
	Short-reads mNGS	MECOS	Short-reads mNGS	MECOS	Short-reads mNGS	MECOS
Total contig length (Mb)	1.8	1.9	3.0	3.1	6.0	5.8
Contig number (≥200 k)	0	11	14	13	0	0
N50 contig length (kb)	46	568	538	918	8	9

	*Salmonella enterica*		*Staphylococcus aureus*			
	Short-reads mNGS	MECOS	Short-reads mNGS	MECOS		
Total contig length (Mb)	4.7	4.8	2.7	2.7		
Contig number (≥200 k)	17	7	7	14		
N50 contig length (kb)	154	1,065	204	516		

We then assessed the ability of MECOS to produce contigs in real samples by employing it on six fecal samples (three from human and three from mouse) (Table S2). This analysis generated 223 M reads on average per sample. We used six short-reads mNGS libraries with comparable number of reads as control (Table 2).

**TABLE 2:** Basic sequencing data statistics and assembly of newly collected samples

	Human					
	H1		H2		H3	
	Short-reads mNGS	MECOS	Short-reads mNGS	MECOS	Short-reads mNGS	MECOS
Total sequenced reads (M)	195	205	879	247	757	186
Input genomic DNA	200 ng	0. 1 ng	200 ng	0. 1 ng	200 ng	0. 1 ng
Total contig length (Mb)	307	216	296	144	376	163
Contig number (≥1 k)	55,600	13,272	39,633	6,216	68,263	9,634
Contig number (≥500 k)	1	36	3	47	3	22
N50 contig length (kb)	14,309	90,483	27,300	183, 192	18,618	69,049
Median co-barcode fragments per contig		365		500		473
Barcode number		34,926,480		40,713,529		33,713,345
Barcode with >1 paired reads		18,722,052		21,879,879		17,443, 183
Barcode with >5 paired reads		7,515,421		9,273,338		6,541,754
Barcode with >10 paired reads		1,766,458		2,312,003		1,411,531
Max co-barcode reads per fragment		184		262		136
Median co-barcode reads per fragment		2		2		2
Mean co-barcode reads per fragment		4		4		2
Bin number	54	109	58	89	65	73
HMG bin number	23	45	34	28	31	31

	Mouse					
	M1		M2		M3	
	Short-reads mNGS	MECOS	Short-reads mNGS	MECOS	Short-reads mNGS	MECOS
Total sequenced reads (M)	185	231	549	230	635	236
Input genomic DNA	200 ng	0. 1 ng	200 ng	0. 1 ng	200 ng	0. 1 ng
Total contig length (Mb)	246	177	706	274	619	213
Contig number (≥1 k)	44,678	9,617	157,551	20,886	146,355	18, 129
Contig number (≥500 k)	1	26	2	29	2	23
N50 contig length (kb)	18,876	103,318	11,061	68,097	10,058	57, 109
Median co-barcode fragments per contig		549		395		419
Barcode number		38,058, 198		38,858,024		39,788,605
Barcode with >1 paired reads		21,067,980		19,947,743		20,602,652
Barcode with >5 paired reads		8,608,296		8, 186,742		8,403,001
Barcode with >10 paired reads		2,012,749		2,211,328		2,252,472
Max co-barcode reads per fragment		260		878		806
Median co-barcode reads per fragment		2		2		2
Mean co-barcode reads per fragment		2		2		2
Bin number	77	135	129	169	121	159
HMG bin number	50	32	70	51	60	38

Similarly, we first detected genus-level community composition and abundance of fecal samples with Kraken (version 1.0), and found that the results were largely concordant between MECOS and short-reads mNGS (Fig. S2A). We also compared the relative read abundance between the methods and found a strong correlation (Fig. S2B). These results suggest that MECOS likely does not introduce biases in real samples.

**Fig 2:**
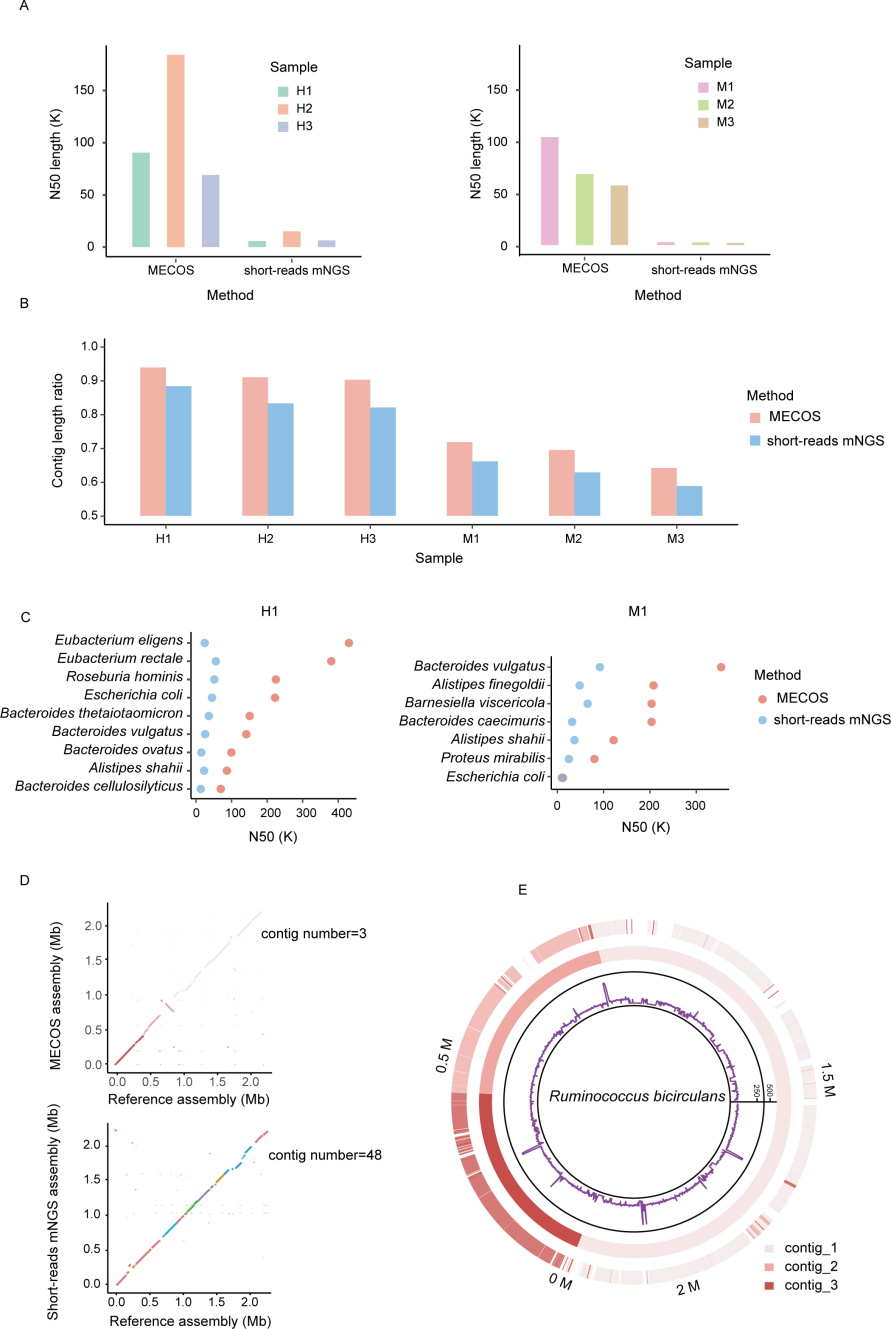
Contig and N50 lengths in different samples. (**A**) Contig N50 length comparison between different workflows employed on human and mouse gut microbiome samples. (**B**) The overall species-belonging contig length to raw contig length ratio. In total, 90% of MECOS assembly contigs of human samples could be assigned to the species level compared to 80% obtained using standard short-reads mNGS approach. (**C**) Contig N50 length of the most abundant species, from H1 and M1. (**D**) Comparisons of representative MECOS and short-reads mNGS genome drafts to reference genomes. Dot-plot alignments between MECOS or short-reads mNGS drafts (y-axis) and the closest available reference genome (x-axis). For each dot plot, specific colors correspond to the alignment of single contigs in the MECOS draft against the available reference genome. (**E**) The most contiguous genome assembly from the human gut sample 1: three contigs cover 83.4% of the longest reference sequence for *Ruminococcus bicirculans*. The three contigs are consistently aligned to the reference genomes with a 170 kb inversion, which is supported by barcode evidence.

We next assembled the reads and obtained 12,959 contigs on average for each of the six samples, with N50 length varying between 57K to 183K, which is significantly larger than those obtained in short-reads mNGS libraries (Fig. 2A). We used Kraken to assign contigs to species, which was possible for 90% and 80% of MECOS and short-reads mNGS contigs, respectively. This indicates MECOS performs better in species-level assignment (Fig. 2B).

Next, we blasted the contigs to reference genomes available from the NCBI RefSeq database. We selected species with >80% coverage as high-quality genomes. The high-quality genomes obtained from human samples were *Eubacterium*, *Bacteroides*, and *Roseburia*, whereas mice samples contained *Bacteroides*, *Alistipes,* and *Barnesiella*. Contig length was higher in MECOS compared to short-reads mNGS in both human and mice samples. For example, the MECOS contig N50 length in *Eubacterium eligens* has over 400 kb, which is 10-fold than that obtained in short-reads mNGS (Fig. 2C; Fig. S3). Crucially, MECOS recovers complete genomes from metagenomic sequencing data. In fact, we recovered the nearly complete genome of *Ruminococcus bicirculans* from one human sample, with three contigs of sizes 1.4M, 0.6M, and 0.5M (Fig. 2D), which covered >83.4% of the total reference genome size (Fig. 2E) (accession number: HF545616.1). The data suggest that MECOS significantly improves genome recovery from metagenomic data.

### MECOS identifies multi-drug-resistant bacteria

**Fig 3:**
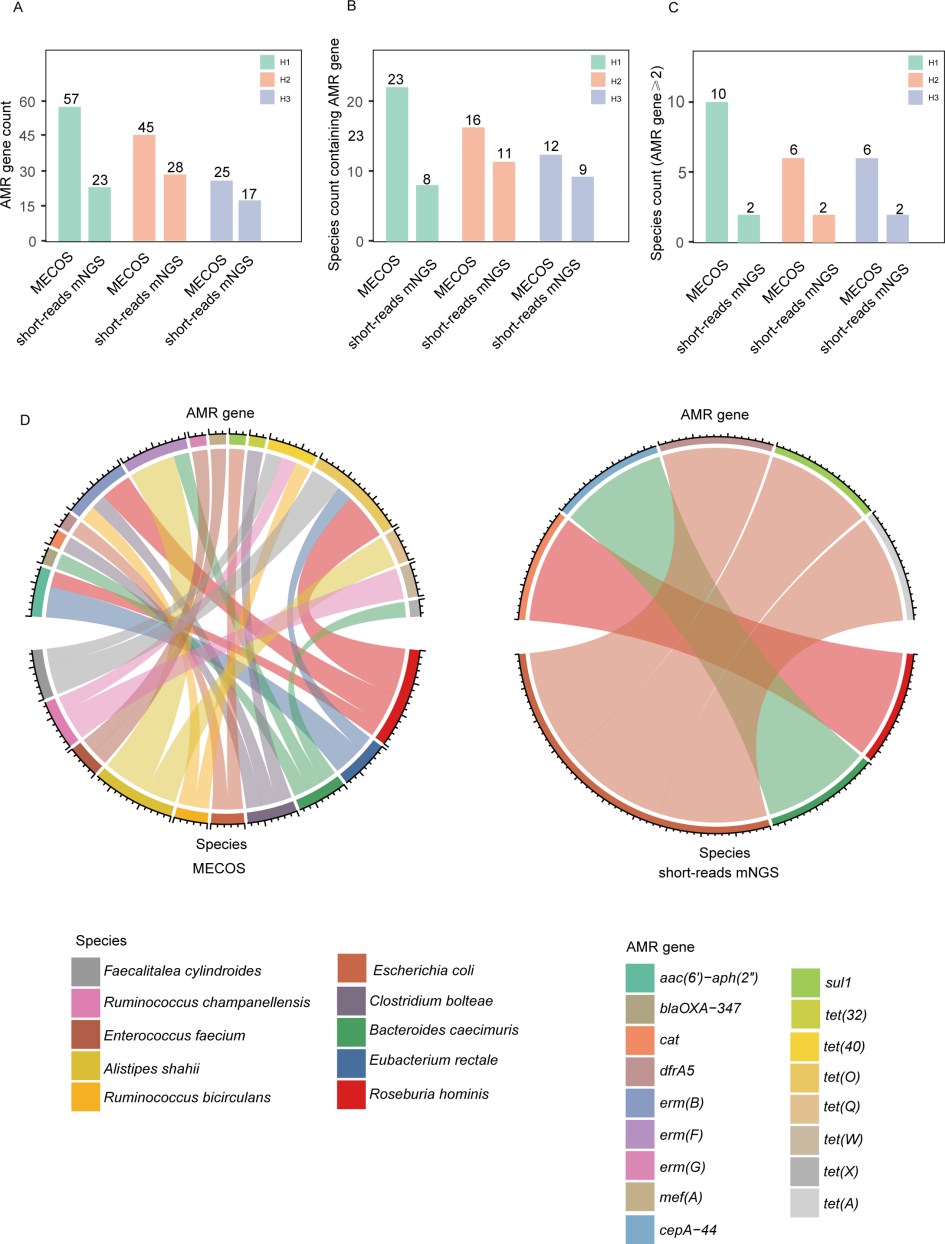
Identification of multiple drug resistance genes in bacteria. (**A**) Comparison of AMR gene count between MECOS and short-reads mNGS from human gut microbiome samples. (**B**) Comparison of species count containing AMR gene between MECOS and short-reads mNGS from human gut microbiome samples. (**C**) Comparison of species count (AMR gene count ≥2) between MECOS and short-reads mNGS from human gut microbiome samples. (**D**) Chord diagram showing drug resistance genes in different species as identified by MECOS and short-reads mNGS.

We compared the ability of MECOS and short-reads mNGS to detect total antimicrobial resistance (AMR) gene count, species count containing AMR gene, and the number of species containing two or more drug resistance genes. We found that MECOS could detect more drug-resistant genes (Fig. 3A). In addition, MECOS was more efficient in assigning genes to bacterial species, enabling the identification of a higher number of multi-drug-resistant bacterial species compared to short-reads mNGS. Specifically, MECOS was able to detect 17 drug-resistant species on average compared to short-reads mNGS’s nine species (Fig. 3B), including a higher number of species (seven in average) possessing more than two AMR genes compared to short-reads mNGS (Fig. 3C). These results suggest MECOS is better suited for identifying multi-drug-resistant bacteria.

In order to gain further insights into the distribution of drug resistance genes in different species, we selected the sample with the highest number of resistance genes (H1) to build a chord diagram. MECOS revealed the presence of drug resistance genes in a total of 10 bacterial species, of which only three were also identified using short-reads mNGS. Moreover, MECOS detected three drug resistance genes in *Clostridium boltae* and *Roseburia hominis*, whereas short-reads mNGS only revealed one gene in the latter species. Accordingly, MECOS has a superior performance identifying multiple coexisting drug-resistant genes in bacteria (Fig. 3D).

### MECOS identified a higher number of horizontal gene transfer events

**Fig 4:**
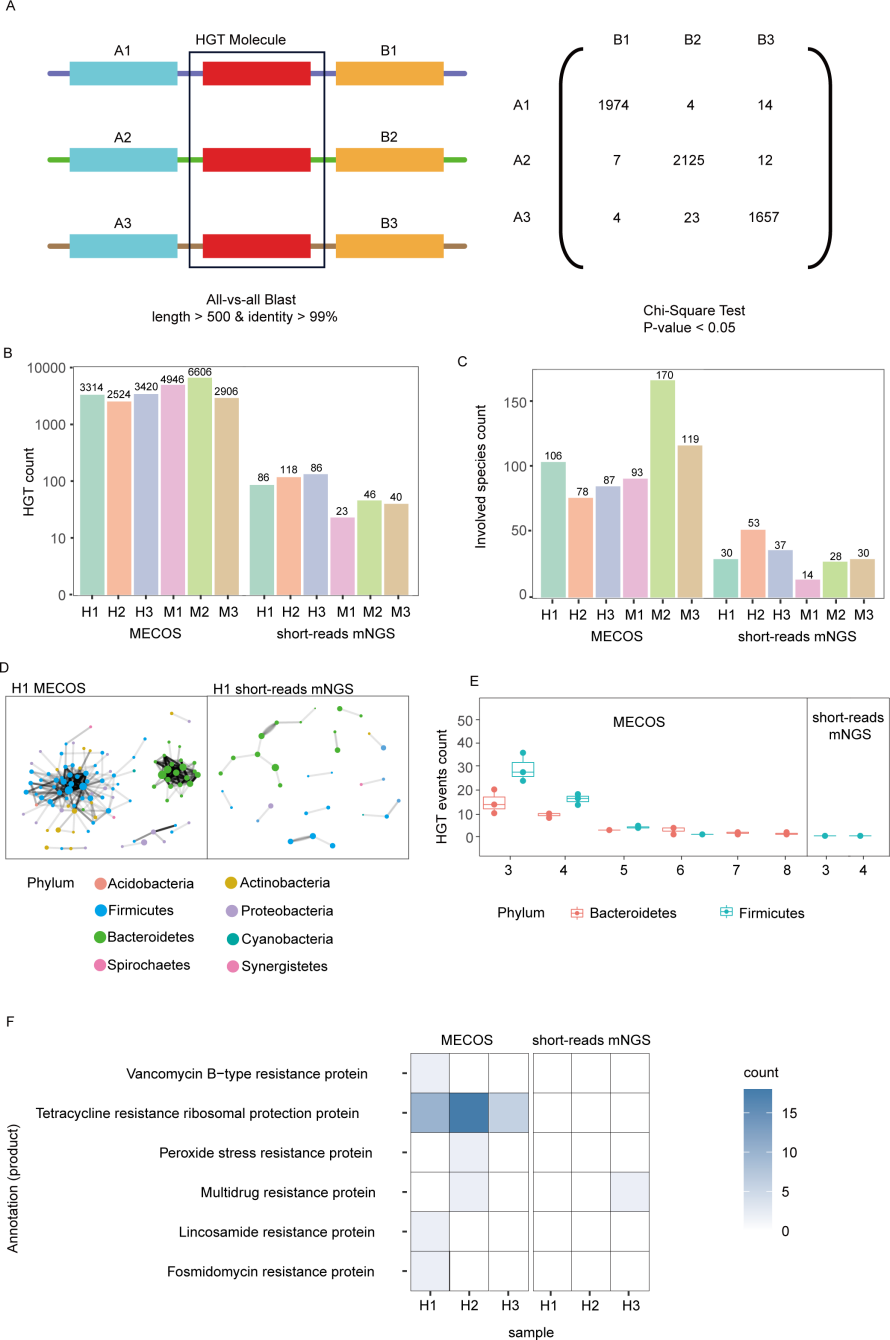
Identification of HGT events in bacteria. (**A**) Definition of HGT events: HGT events between genomes from different species were defined by the presence of a common sequence of at least 500 bp with 99% identity. χ^2^ tests were performed for each HGT event with its multi-alignment. Only predicted events passing the χ^2^ tests were kept for further analysis. Despite this conservative approach, we found significantly more candidates using MECOS than short-reads mNGS. (**B**) Comparison of the number of HGT events predicted by MECOS and short-reads mNGS from gut microbiome samples showed that MECOS detects more HGT events. (**C**) Comparison of the species involved shows MECOS detects a higher number of species. (**D**) Network of HGT events constructed by MECOS and short-reads mNGS of H1. HGT events are mainly concentrated in Bacteroidetes and Firmicutes. (**E**) Distribution of the number of species in which HGT events are predicted by both MECOS and short-reads mNGS showed that eight species participated in MECOS-predicted HGT events compared to four species predicted by short-reads mNGS. (**F**) The detection of functional regions associated with drug resistance and virulence using MECOS and short-reads mNGS.

MECOS had the capability to acquire both long contigs and a large number of co-barcode information, which may enhance the ability of HGT events detection. We evaluated the effectiveness of MECOS in identifying HGT events by building a model and defining a mobile sequence as two similar sequences present on contigs of different species and that met specific criteria. Specifically, we required at least 99% sequence identity and fragments longer than 500 bp (Fig. 4A left). Subsequently, we conducted a validation step to confirm candidate HGT events by assessing the presence of co-barcodes between the upstream and downstream regions of each mobile candidate. In addition, we performed a χ^2^ test in each candidate region to confirm that the linkage observed between the upstream and downstream sequences adjacent to the mobile candidate was not randomly occurring within the multi-alignment (Fig. 4A right, Methods).

We examined the presence of the 1,919 bp gene *tet(O*) in both *Eubacterium eligens* and *Streptococcus pyogenes* to validate the identification of HGT events using MECOS. Despite being present in different genera, the gene exhibited 99.35% sequence identity, and thus very likely resulted from an HGT event. The presence of this HGT event was confirmed by detecting the same MECOS co-barcode in adjacent regions in both species (Fig. S4A), as well as by PacBio reads (Fig. S4B) covering the region.

We applied the HGT model to calculate HGT events in the MECOS and short-reads mNGS libraries. The MECOS library, which retains long-fragment DNA information resulting in longer assemblies, detected a significantly higher number of HGT events compared to the short-reads mNGS library. Taking H1 as an example, 3,314 HGT events involving 106 species were identified in MECOS, with 22 species belonging to Bacteroidetes and 48 species to Firmicutes. In contrast, the short-reads mNGS library identified only 86 HGT events involving 30 species, 9 of which belonged to Bacteroidetes and 11 to Firmicutes (Fig. 4B and C) (Tables S3 and S4). The MECOS library detected approximately 40-fold HGT events compared to the short-reads mNGS library. We identified most HGT events within Bacteroidetes and Firmicutes, which is in line with previous single-cell genomics sequencing research (19). Finally, HGT events predominantly occurred within a single phylum (Fig. 4D). These results suggest the MECOS library is more effective in identifying HGT events from metagenomic data than the short-reads mNGS library.

We then examined MECOS-inferred HGT events involving more than two species in H1 and found 97 HGT events in Bacteroidetes and 153 HGT events in Firmicutes. Of these, only three HGT in Firmicutes involving more than two species were detected through short-reads mNGS. Finally, MECOS detected HGT events shared by up to eight species, while short-reads mNGS could only detect events shared by at most four species (Fig. 4E).

In order to investigate the functional impacts of HGT events, we annotated the contigs of all assemblies, identifying mobile candidates overlapping with at least 50% of a coding region. Our analysis revealed that the genes overlapping with HGT regions are involved in gene replication, recombination, and repair (Fig. S5). We further annotated HGT regions obtained by MECOS utilizing a drug resistance database, and discovered a significantly higher number of drug resistance genes encoding for tetracycline resistance (Fig. 4F). These results demonstrate that MECOS can identify drug resistance events using a metagenomics approach.

## DISCUSSION

HGT is a fundamental evolutionary mechanism that facilitates the direct transfer of genetic material between distantly related species, enabling bacteria to acquire novel traits, including antibiotic resistance and pathogenic toxins (20). Current bioinformatics methodologies primarily rely on identifying past HGT events by analyzing phylogenetic trees or inconsistencies in genome composition. However, these methods necessitate the availability of complete and thoroughly annotated genomes and sufficiently large genetic variation to enable the accurate detection of HGT events.

In this study, we developed MECOS, a new co-barcoding sequencing system to identify recent HGT events. The computational resources required for MECOS are comparable to those needed for short-reads mNGS (21). Importantly, MECOS requires only 0.1 ng high-quality DNA, which is much lower compared to the 200 ng required for short-reads mNGS (Table 2). Several short-reads mNGS kits have been developed to accommodate low input DNA, comparable to or even lower than MECOS. However, it has been observed that such short-reads mNGS kits designed for low input DNA often necessitate an increased number of PCR cycles. This rise in PCR cyclic number could subsequently lead to higher duplication rates and adversely impact the sequencing coverage. In addition, MECOS is not expensive, the overall cost for MECOS was just twice as much compared to the short-reads mNGS used in our study. Moreover, we found that barcoded reads circumvent conventional short-read sequencing issues by providing longer contigs that carry long range information on DNA fragments, and facilitate downstream analyses, such as the detection of multi-resistance bacteria and HGT events.

Furthermore, MECOS was able to detect a higher number of drug-resistant genes associated with HGT events in each species compared to short-reads mNGS. The typical length of a HGT-associated drug resistance gene is approximately 1,000 bp, with the number of contigs > 500 kb obtained by MECOS exceeding that of short-reads mNGS on average by 15-fold (Table 2). MECOS can thus detect a higher number of drug resistance genes on individual contigs, thereby facilitating the identification of multi-resistant bacterial strains.

It is recognized that HGT events are difficult to detect as the exact genomic breakpoints are usually unknown. We identified 50-fold more HGT events in MECOS compared with short-reads mNGS, as a result of the longer contigs generated with the new approach, circumventing difficulties in assembly of traditional short-reads mNGS techniques (Fig S6). While longer contigs have successfully addressed the issue of genetic breakpoints and have indeed achieved significant progress in detecting HGT events, assembly errors may arise during the assembly process of these longer contigs, resulting in the identification of erroneous HGT events. The MECOS not only enables the acquisition of longer contigs but also provides access to over 30 million co-barcode information (Table S2). Leveraging this co-barcode information, we can conduct a χ^2^ test on the HGT results to correct the potential wrong HGT events. We observe that HGT events were enriched in gene replication, recombination, and repair regions, helping to demonstrate the reliability and robustness of our strategy. MECOS offers novel ways to examine the interplay between multiple microorganisms within the gut microbiome.

The HGT events detected overlapped regions of tetracycline resistance and ribosomal protection, which is consistent with previous studies. Tetracycline is an inexpensive broad-spectrum antibiotic widely used to treat bacterial infections in humans and animals. The overuse of tetracycline increases the likelihood that this antibiotic is present in foods of animal origin and the chances of emergence of resistance bacteria in healthy human gastrointestinal tract (22).

The present study still has certain limitations. While MECOS serves as a novel co-barcoding method and indeed exhibits several advantages compared to conventional second-generation sequencing technologies, there are still areas that need improvement. Specifically, the improvements in N50 and the quality of metagenome-assembled genomes are relatively lower. These limitations can be attributed to insufficient DNA fragment lengths and suboptimal insertion density of the modified Tn5 transposome. Addressing these issues represents a key area for future research and development.

In conclusion, we developed a co-barcoding approach (MECOS) to assemble gut metagenomes for three healthy human and three normal mice samples. Compared to short-reads mNGS approaches, we detected more numerous and more accurate HGT events using MECOS, which are associated with antibiotic resistance.

## MATERIALS AND METHODS

### Sample collection and long DNA extraction

Fecal samples were collected using the MGIEasy Fecal Sample Collection Kit (PN: 1000005265), immediately stored in 2 mL stabilizer, transported to the lab in 24 h, and stored at −80°C. Microbial Community Standard was obtained from ZymoBIOMICS (D6300). High molecular weight DNA was extracted using the MagAttract HMW DNA Kit (Qiagen, Cat. No. 67563) following the instructions of the manufacturer. Before using the kit for extraction, the sample was mixed with 200 µL lysozyme (200 mg/mL) at 37°C for 1 h. The Qubit dsDNA HS Kit was used to quantify DNA, and purity was determined by NanoDrop. The integrity of DNA was analyzed using 130 V electrophoresis for 90 min on a 0.8% 1× TAE (Tris base, acetic acid, and ethylenediaminetetraacetic acid) agarose gel.

### MECOS library preparation and sequencing

The reagents utilized in MECOS were procured from the MGI stLFR library preparation kit (PN: 1000021745). However, we made experimental modifications to enhance their suitability for co-encoding sequencing of microorganisms. The whole workflow employed for MECOS was illustrated in Fig. 1A, and included the following steps: (i) transposome insertion; (ii) co-barcoding of long fragments; (iii) ligation and PCR; and (iv) DNA Nanoball (DNB) generation and sequencing. Specifically, the process started with the insertion of the transposome and sequence hybridization in long genomic DNA molecules approximately every 200–2,000 bp. The transposome contained the Tn5 transposase enzyme and synthetic transposon sequence. The Tn5 transposon consisted a single-stranded region designed for hybridization with beads and a double-stranded sequence recognized by the transposase enzyme to facilitate the transposition reaction. The transposome integrated DNA was then mixed with the beads. The surface of the beads contained an adapter sequence comprising a common PCR primer site. A unique bead barcode sequence was 42 bp, with a specific structure comprising a 10 bp barcode sequence, followed by a 6 bp fixed sequence, another 10 bp barcode sequence, an additional 6 bp fixed sequence, and concluding with a final 10 bp barcode sequence. There were a total of 1,536 different sequences (Table S1) for each 10 bp barcode sequence, resulting in a grand total of 3,623,878,656 distinct bead barcode sequences (calculated as 1,536 * 1,536 * 1,536). In addition, the beads contained a capture sequence complementary to the hybridization sequence on the integrated transposase, which captured the DNA inserted into the transcriptome. In our case, the bead barcode library contained 3.6 billion unique barcodes and each surface included ~400,000 copies of the same bead barcode sequence. The beads made it possible to capture long-fragment DNA by ligating the adapters to the integrated transposons, after which the DNA/transposase complexes were disrupted into sub-fragments smaller than 2,000 bp. The sub-fragments from the same original long DNA fragment carried identical barcodes. Then, we continued the next library-processing steps, including the ligation of a second adapter, PCR and PCR-product purification. Finally, we sequenced the barcoded sub-fragments using 100 bp paired-end reads on a MGISEQ-2000RS High-throughput Sequencing Set (PN:1000011545). The sequencing conditions were set as dual barcode sequencing method, barcode length of 42 bp, dual barcode length of 10 bp, and read length of 100 bp.

### Short-reads mNGS library preparation and sequencing

The extracted DNA was fragmented ultrasonically with Covaris E220 (Covaris, Brighton, UK), yielding 300 to 700 bp fragments. The short-reads mNGS library was constructed using the MGIEasy Universal DNA Library Prep Set (PN:1000006985) and sequenced using 100 bp paired-end reads on a MGISEQ-2000. After removing low-quality reads using a revised version of fastp (23), high-quality reads were aligned to hg19 using Bowtie (24) to remove human reads (identity ≥0.9).

### PacBio SMRT bell library preparation and SMRT sequencing

The PacBio SMRT sequencing was started using the PacBio Template Prep Kit (Pacific Biosciences, Cat NO,101–357-000) to generate SMRTbell libraries, which was created by ligating hairpin adapters to double-stranded DNA, thereby circularizing them into a construct termed a SMRT bell. Then, the SMRTbell libraries were sequenced with the Pacific Sequel platform (Pacific Biosciences, Cat NO, 101–310-500). A primer and a polymerase were annealed to the adapter and loaded into SMRT Cell. Each SMRT Cell contained millions of nanoscale observation chambers called Zero Mode Wave guides, where a single DNA polymerase attached to a single DNA template was anchored to the bottom and the polymerase continuously incorporated fluorescently labeled nucleotides to emit fluorescence signals, while the camera recorded in real time to realize simultaneous synthesis and sequencing.

### Co-barcode assembling

The metagenomic sequencing data underwent several processing steps to obtain a comprehensive assembly of the metagenome. Initially, the short reads were stripped of their barcodes and assembled using the MetaSPAde (25) assembler (version 1.1.3) with default parameters. This step produced a set of initial sequence contigs, which serve as the starting point for further assembly. The next step involved the Athena assembler (26) (version 1.3). The seed contigs obtained from MetaSPAde were fed into Athena for additional metagenome sequence assembly. The same barcoded short reads were mapped back to the seed contigs using the BWA-MEM function (version 0.7.15) with default parameters. The read pairs that span across different contigs were used to create edges in a scaffold graph. At each edge of the scaffold graph, Athena utilized the short-reads mNGS and their attached barcodes to propose a simplified subassembly problem. The resulting intermediary subassembled contigs, along with the initial seed contigs, were then treated as reads and passed on to the long-read De Bruijn graph-based assembler, Flye (version 2.3.1). Flye used this combined set of much longer contigs to determine the optimal assembly of the target genome, thereby improving the overall quality and completeness of the metagenome assembly.

### Species annotation on contigs

The initial step involved assigning the contigs using Kraken (version 1.0), a taxonomic classification tool, to identify related species and select them as candidate species. Next, we performed BLAST(27) (version 2.5.0+) analysis of the contigs against the NCBI RefSeq database to obtain additional information for annotation. Specifically, species with a coverage greater than 80% were considered as high-quality genomes and retained for further analysis.

### Genome draft binning and quality control

Raw contigs were subsequently binned with MetaBAT2 (version 2.12.1) to form genome drafts. Bins were then evaluated with CheckM (version 1.2.0) for completeness and contamination as genome grafts. Specifically, we defined high- or medium-quality genomes (HMGs) as those with over 50% completeness and less than 5% contamination. Subsequently, the identified HMGs underwent taxonomic identification using GTDB-tk (version 1.7.0) with the Genome Taxonomy Database (r202) to assign taxonomic classifications.

### Gene prediction and functional annotation

We annotated the genes of the species in contigs with Prokka (v1.14.5) (28) The functions of non-redundant genes were annotated using both Cluster of Orthologous Group (COG) and Kyoto Encyclopedia of Genes and Genomes (KEGG) pathway databases with eggnog-mapper (29, 30) (version 2.0, parameters: e < 0.0001). We screened for antibiotic resistance genes using ABRicate and ResFinder (31) on the Comprehensive Antibiotic Resistance Database (32).

### Identification of horizontal gene transfer events

We detected HGT events by searching mobile sequence candidates shared by two contigs belonging to different species or on the same evolutionary path. Firstly, we performed all-to-all blast in all contigs. Then, we searched for mobile sequences >500 bp and >99.00% identical in highly similar metagenomes that often cause short-read mis-assemblies. Co-barcoded reads contained long-range information on DNA sequences that may encompass mobile elements. Specifically, when the reads were mapped to contigs, the closer the two unique regions were, the more barcodes they shared. Hence, this strategy also consisted of a multi-alignment between contigs. The rationale was that in multi-alignment mobile sequenced, any upstream unique region should share the majority of barcodes with downstream unique regions on the same contig, but not with other contigs. The level of sharing between up- and down-stream regions around a mobile sequence of the multi-alignment was measured using a χ^2^ test. For each contig in the multi-alignment, we calculated the same contig to overall sharing ratio. If the expected value was <5, we sequentially discarded small ratio contigs until the expected value was >5 or there was only one contig left. Only the mobile sequence passing the χ^2^ test were kept for further analysis.

## Data Availability

The sequencing data have been submitted to the National Genomics Data Center, China National Center for Bioinformation, and are publicly available with accession no. CRA009846. The original code is available in github. All data are available in the main text or the supplemental material.

## SUPPLEMENTAL MATERIAL

Figures S1 to S6, Tables S1, S2, and S4 spectrum.03602-23-s0001.docx Supplementary results, figures, tables.

Table S3 spectrum.03602-23-s0002.xls Detailed information for the HGT events.

ASM does not own the copyrights to Supplemental Material that may be linked to, or accessed through, an article. The authors have granted ASM a non-exclusive, world-wide license to publish the Supplemental Material files. Please contact the corresponding author directly for reuse.
