# Supplementary material for: Detecting horizontal gene transfer with metagenomics co-barcoding sequencing
Source: Microbiol Spectr. 2024 Feb 5;12(3):e03602-23. doi: 10.1128/spectrum.03602-23 (PMC10913427; doi:10.1128/spectrum.03602-23)
Supplement: Figures S1 to S6, Tables S1, S2, and S4 — Supplementary results, figures, tables. [file spectrum.03602-23-s0001.docx]

**Supplemental information**

**Detecting horizontal gene transfer with metagenomics co-barcoding sequencing**

Kai Han, Jiarui Li, Duo Yang, Qinghui Zhuang, Hui Zeng, Chengbo Rong, Jinglin Yue, Na Li, Chaoyang Gu, Liang Chen and Chen Chen

**Detecting horizontal gene transfer with metagenomics co-barcoding sequencing**

Kai Han,^a^ Jiarui Li,^a^ Duo Yang,^a^ Qinghui Zhuang,^a^ Chengbo Rong,^a^ Jinglin Yue,^a^ Na Li,^a^ Chaoyang Gu,^a^ Liang Chen^a^ and Chen Chen^a^#

^a^ Biomedical innovation center and Beijing Key Laboratory for Therapeutic Cancer Vaccines, Beijing Shijitan Hospital, Capital Medical University,Beijing, China

#Address correspondence to Chen Chen, [chenchen1@ccmu.edu.cn](mailto:chenchen1@ccmu.edu.cn) (C.C.)

Kai Han and Jiarui Li contributed equally to this work.

**This PDF file includes:**

**Supplementary results, figures, tables and references**

Figures S1 to S6

Table S1, S2 and S4

**SUPPLEMENTARY RESULTS**


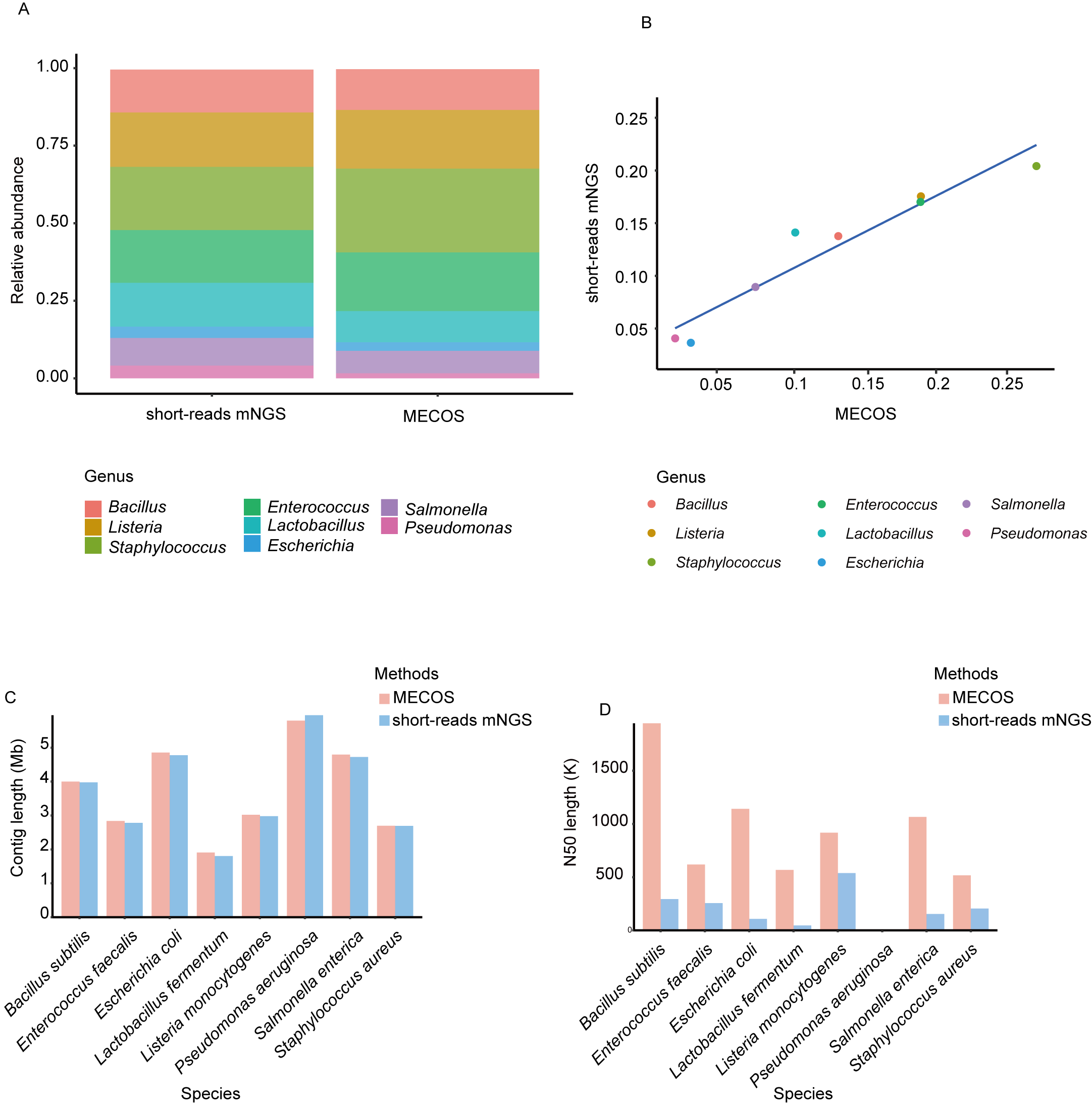


Figure S1. (A) Relative abundance of 8 bacterial species in Microbial Community Standard, the relative representation appears fairly concordant among the two different library-preparation methods (MECOS and Short-reads mNGS). (B) Relative read abundance comparison between MECOS reads and Short-reads mNGS reads for Microbial Community Standard. The correlation is high. (C) The total length of contigs assigned to species among the two different library-preparation methods (MECOS and Short-reads mNGS) for Microbial Community Standard. (D) Contig N50 length of different species in Microbial Community Standard.


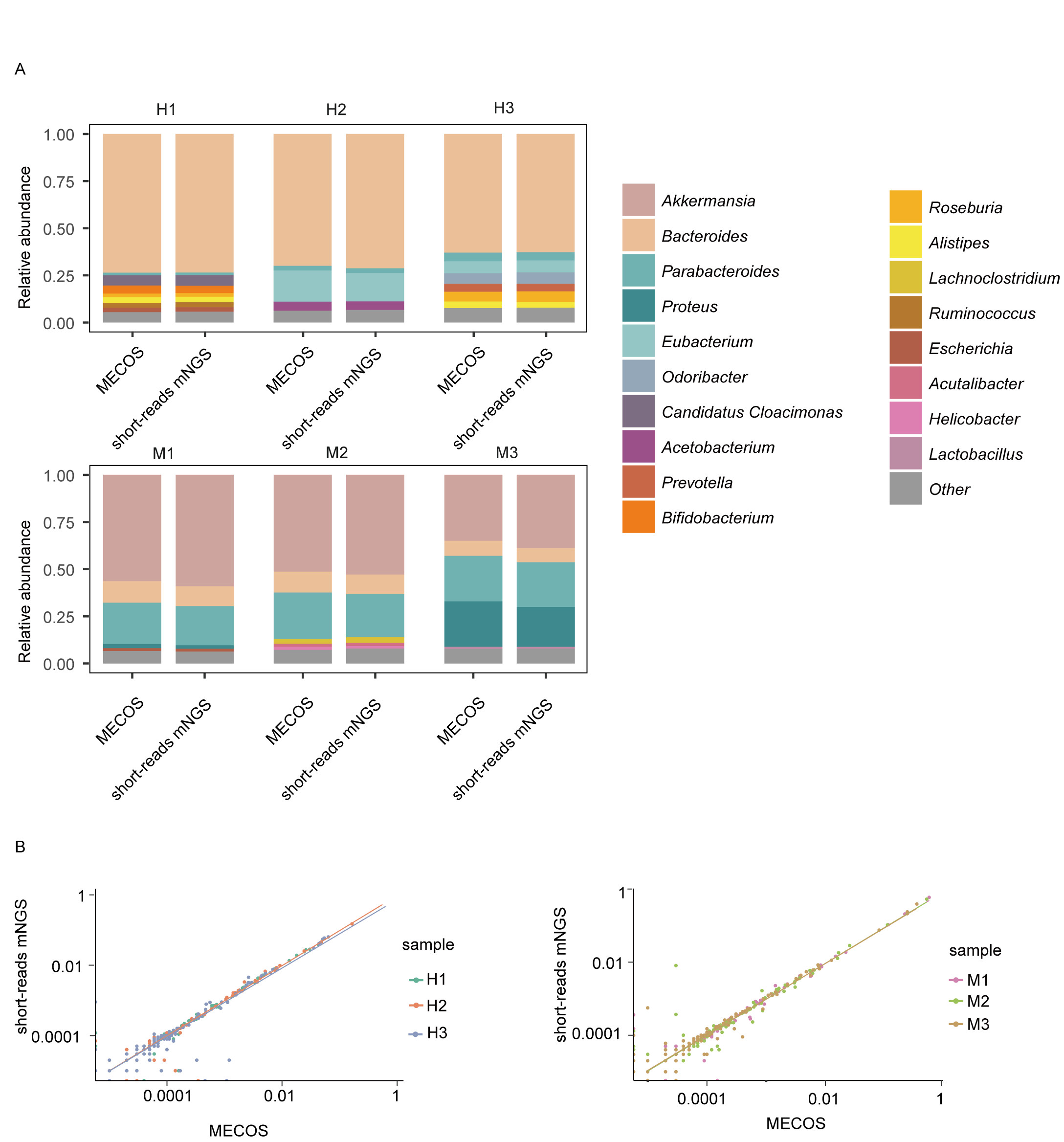


Figure S2. (A) Relative abundance of genera, as determined by short-read classification for each of the three libraries from samples human and mouse. The relative representation of genera appears fairly concordant among the two different library-preparation methods (MECOS and Short-reads mNGS) for each sample.(B) Relative read abundance comparison between MECOS reads and Short-reads mNGS reads for each sample at genus level. The correlation is high.


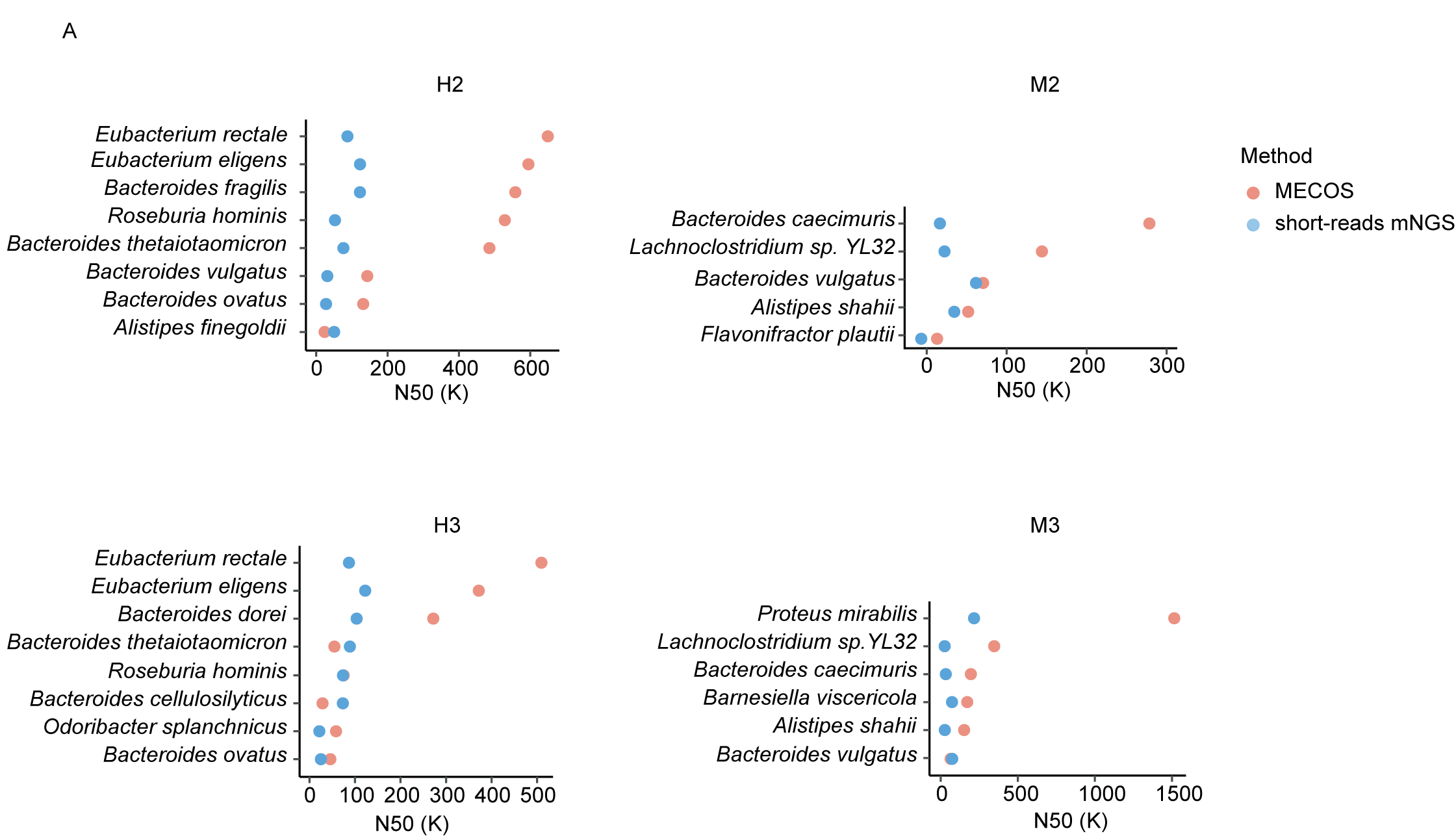


Figure S3. (A) Contig N50 length of the most abundant species from others 4 samples.


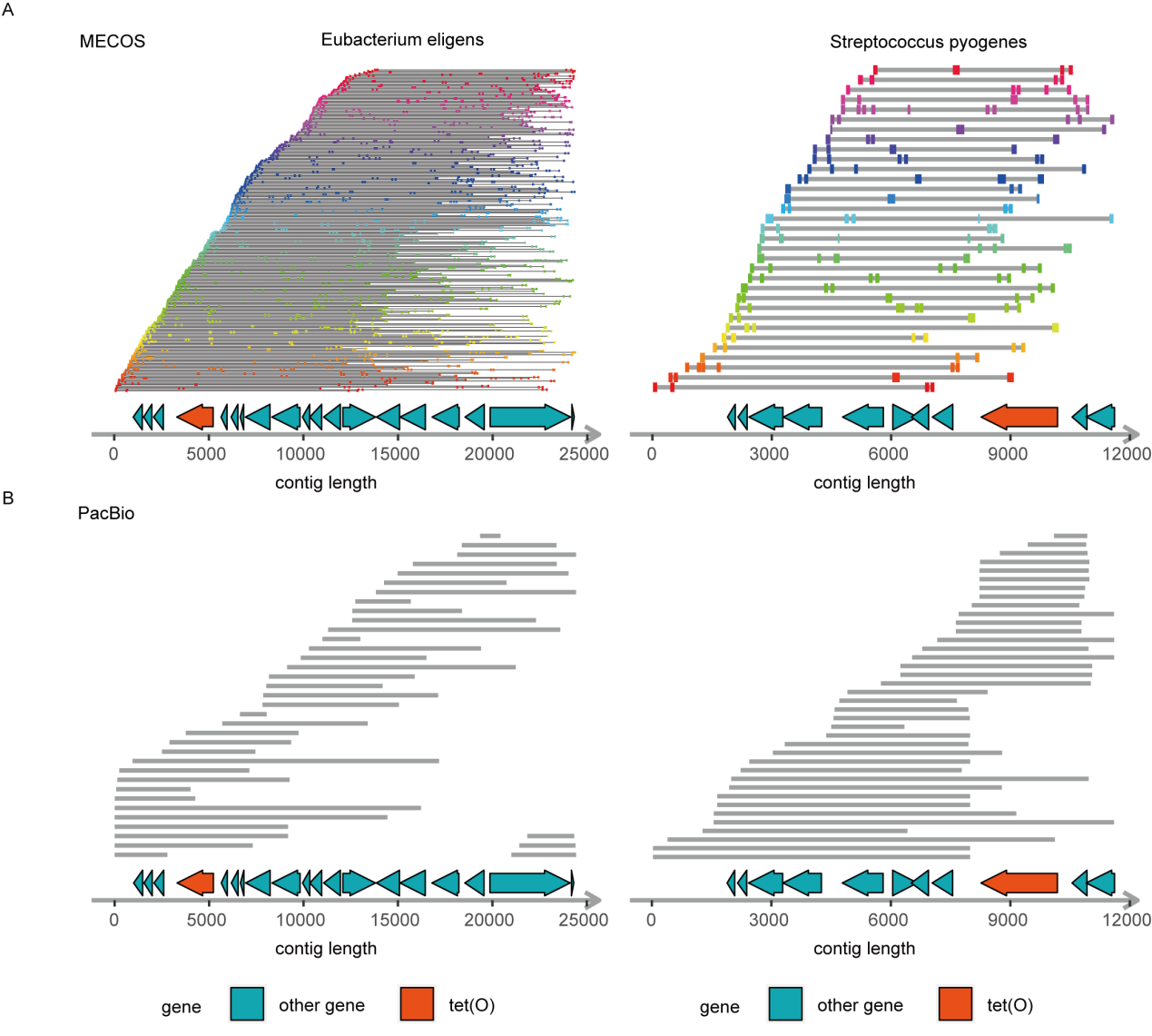


Figure S4. (A) Tet(O) gene HTG event recognition in *Eubacterium eligens* and *Streptococcus pyogenes* through MECOS. (B) Tet(O) gene HTG event recognition in *Eubacterium eligens* and *Streptococcus pyogenes* through PacBio.


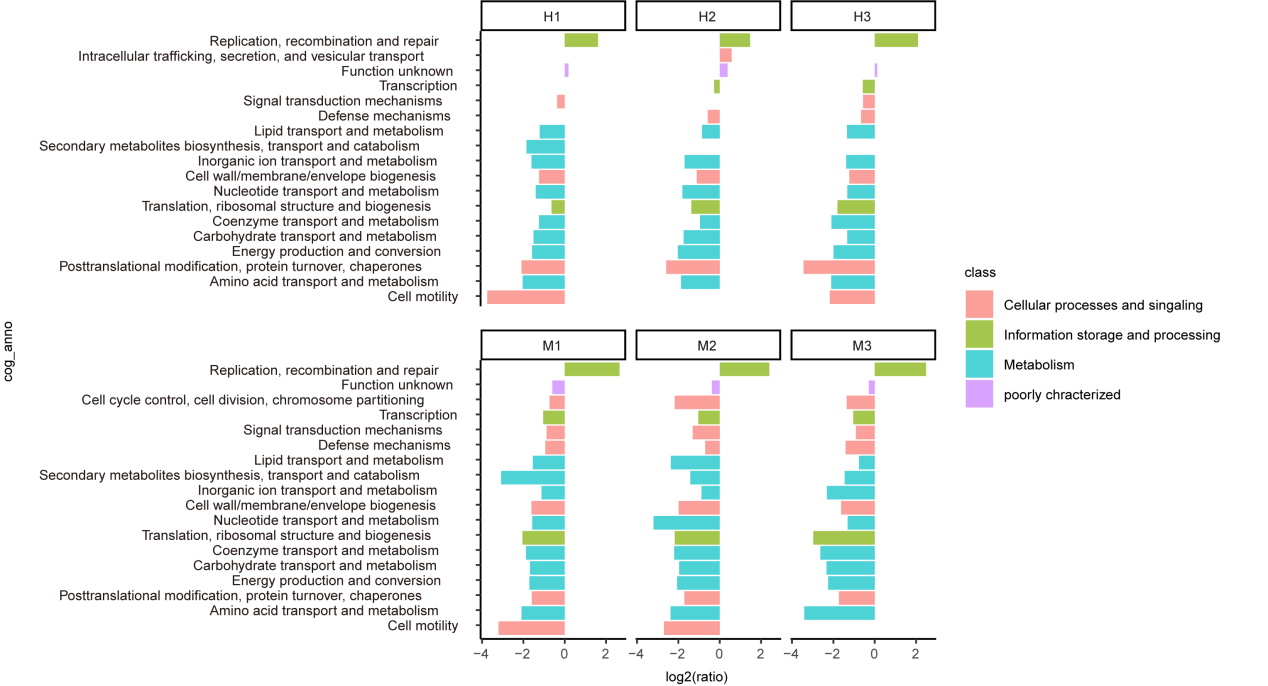


Figure S5. The results of a comparison between the functional categories assign to genes involved in horizontal gene transfer (HGT) events (n= 24,756) and those of other genes (n= 971,352). Significance is determined by a Fisher-exact test, with a significance level adjusted to a p-value below 0.05 and further corrected for multiple comparisons using false discovery rate (FDR) correction. Functional categories with a positive effect size indicate over-representation in the HGT genes.


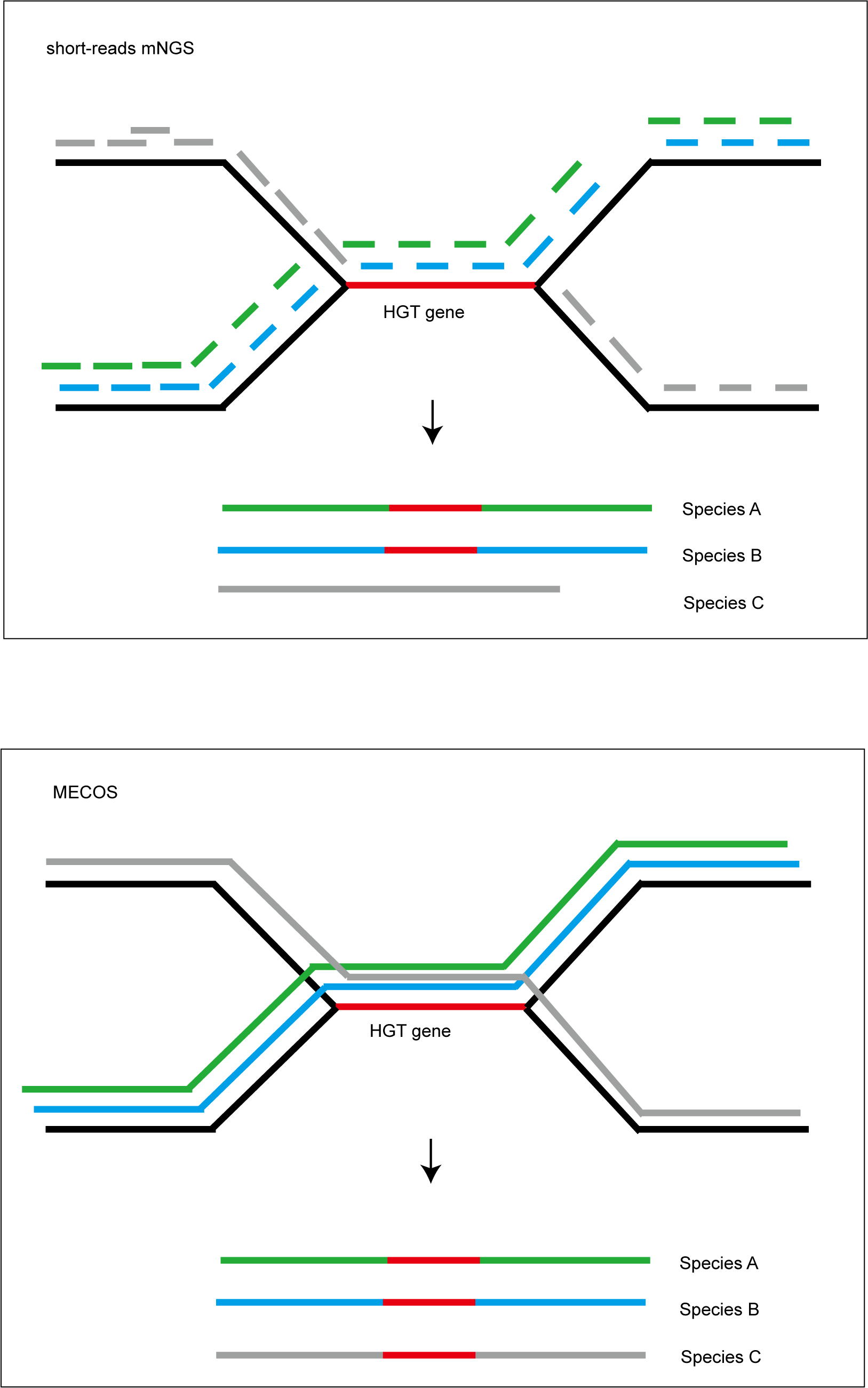


Figure S6. The reads are assembled to contigs through MECOS and Short-reads mNGS, in which reads repeats are also accurately placed. A HGT gene example is shown in red.

Table S1 1536 different barcode sequence

Table S2 The information for human and mouse samples

| Human | Sex | Age | Use of antibiotics within half a year | Underlying diseases (hypertension, Diabetes, Hyperlipidemia) |
| --- | --- | --- | --- | --- |
| H1 | female | 25 | No | No |
| H2 | female | 25 | No | No |
| H3 | female | 27 | No | No |
|  |  |  |  |  |
| Mouse | Mouse strain | Age | Sex | Weight |
| M1 | c57 | 4-6W | male | 18-20g |
| M2 | c57 | 4-6W | male | 18-20g |
| M3 | c57 | 4-6W | male | 18-20g |

Table S3. The detailed information for the HGT events.

Table S4. The information of HGT count and involved species count.

| Sample | HGT count | Involved species count | Sequencing method |
| --- | --- | --- | --- |
| H1 | 3,314 | 106 | MECOS |
| H2 | 2,524 | 78 |  |
| H3 | 3,420 | 87 |  |
| M1 | 4,946 | 93 |  |
| M2 | 6,606 | 170 |  |
| M3 | 2,906 | 119 |  |
| H1 | 86 | 30 | Short-reads mNGS |
| H2 | 118 | 53 |  |
| H3 | 133 | 37 |  |
| M1 | 23 | 14 |  |
| M2 | 46 | 28 |  |
| M3 | 40 | 30 |  |

**MECOS library preparation protocol**

1. **Tn5 insertion**
   1. Dilute the long genomic DNA molecule to 1-3 ng/μL with TE Buffer, 10 ng gDNA is added to a new 0.2mL PCR tube and supplemented with Molecular Grade Water to 36.8 μL.
   2. Dilute SamBarTIE.
      1. Add 6μL TE Buffer and 2μL SamBarTIE into a new 0.2mL PCR tube, vortex briefly to mix.
      2. transfer 6μL mixed SamBarTIE diluent to another 0.2mL PCR tube and add with 18μL TE Buffer, vortex briefly to mix, labeled as SamBarTIE working fluid.

Note：Because SamBarTIE with the same number has the same Sample Barcode sequence and cannot be sequenced in the same lane，different samples which want to be sequenced in the same lane should select different SamBarTIE.

- 1. Add 10 μL TI Buffer and 3.2μL SamBarTIE working fluid to sample gDNA of Step 1.1, slowly blown and mixed for 10 times with a wide-mouth suction, then incubate for 10 min at 55 ℃.

1. **Co-barcoding of long fragment**
   1. Pipet 0.5μL (0.1ng DNA) Step 1.3 product into a new 0.2mL PCR tube, then add 49.5μL TE Buffer and flick or reverse mix 10 times.
   2. A volume of 30 μL Capture Beads are prepared by washing with Wash Buffer I and suspended in 50μL Capture Buffer V2, then added to 2.1 sample, gently reverse 10 times for mixing and incubate at 60 ℃for 10 min and at 45 ℃ for 50 min.
2. **Ligation reaction Ⅰ**
   1. Add 26 μL Ligation Buffer I V2 and 4 μL DNA Ligase to 2.2 product, incubate for 1 h at 25 °C in 130 μL reaction volume.
   2. Place the product of 3.1 on the magnetic rack for 1~2 min until the liquid was cleared, then remove the supernatant carefully.
   3. Keep the PCR tube on the magnetic rack, add 180 μL Wash Buffer II into the tube, and rotate twice repeatedly the PCR tube 180° in order to let Capture Beads run in the Wash Buffer II and fully clean the beads, then remove all the supernatant completely.
3. **Digestive reaction 1**
   1. Remove the 3.3 PCR tube from Magnetic Rack and add 95 μL Digestion Buffer I and 5 μL Digestion Enzyme, gently reverse about 10 times and remix the beads, then incubate for 10 min at 37 °C.
   2. Immediately add 11 μL TIS Buffer to 4.1 Digestive Reaction 1 product, vortex briefly to mix, then incubate for 10 min at room temperature (20°C to 25°C).
   3. Place the product of 4.2 on the magnetic rack for 1~2 min until the liquid is cleared, then remove the supernatant carefully.
   4. Keep the PCR tube on the magnetic rack, add 150 μL Wash Buffer II into the tube, vortex vigorously for 10 s, then remove the all supernatants completely.
   5. Repeat steps 4.4.
4. **Ligation reaction Ⅱ**
   1. Remove the 4.5 PCR tube from magnetic rack and add 20 μL Pre Ligation Buffer and 4 μL Pre Ligation Enzyme, vortex briefly and remix the beads, then incubate for 30 min at 37 °C.
   2. Add 48μL Ligation Buffer II and 10μL DNA Ligase and 18μLAdapter to 5.1 product, incubate for 2 h at 25 °C in 100 μl reaction volume.
   3. Add 80 μL Wash Buffer II to the product of 5.2, and place it on the magnetic rack for 1~2 min until the liquid was cleared, then remove the supernatant carefully.
   4. Keep the PCR tube on the magnetic rack, add 180 μL Wash Buffer II into the tube, and rotate twice repeatedly the PCR tube 180° in order to let Capture Beads run in the Wash Buffer II and fully clean the beads, then remove the all supernatants completely.
5. **PCR**
   1. Remove the 5.4 PCR tube from magnetic rack and add 75 μL PCR Enzyme mix and 7.5 μL PCR Primer mix and 67.5 μL PCR Molecular Grade Water, vortex briefly and remix the beads.
   2. PCR amplification is performed according to the reaction conditions in Table 1.

Table 1 PCR amplification reaction conditions

| Temperature | Time | Cycle |
| --- | --- | --- |
| 98℃ | 3 min | 1 |
| 95℃ | 30 s | 9 |
| 58℃ | 30 s |  |
| 72℃ | 2 min |  |
| 72℃ | 10 min | 1 |
| 4℃ | Hold |  |

- 1. Place the PCR amplification product of 6.2 on the magnetic rack for 1~2 min until the liquid was cleared, then remove all the supernatant with PCR product carefully to a new 1.5mL EP tube, discard the original PCR tubes with dried Capture Beads.

1. **PCR product purification**
   1. Add 100 μL DNA Clean Beads to the PCR product of 6.3, vortex briefly and remix the beads, then incubate at room temperature for 10 min.
   2. Place the tube on the magnetic rack for 1~2 min until the liquid was cleared, then remove the supernatant carefully.
   3. Keep the tube on the magnetic rack, add 500 μL freshly prepared 80% ethanol rinsed magnetic beads and tube walls, then remove the all supernatants completely.
   4. Repeat steps 7.3.
   5. Keep the tube on the magnetic rack, open the tube cover, and dry the DNA Clean Beads at room temperature for about 3-5 minutes.
   6. Remove the tube from the magnetic rack, add 33 μL TE Buffer for DNA elution, and blow with pipette at least 10 times or vortex until the DNA Clean Beads is completely mixed, then incubate at room temperature for 10 min.
   7. Place the tube on the magnetic rack for 1~2 min until the liquid was cleared, then transfer the supernatant with the barcoded sub-fragments DNA to a new 1.5mL EP tube, discard the original tube with dried DNA Clean Beads.
   8. The barcoded sub-fragments should be stored in -20°C refrigerator and then sequenced on MGISEQ-2000RS High-throughput Sequencing Set.
